# Using latent class analysis to model prescription medications in the measurement of falling among a community elderly population

**DOI:** 10.1186/1472-6947-13-60

**Published:** 2013-05-25

**Authors:** Patrick C Hardigan, David C Schwartz, William D Hardigan

**Affiliations:** 1 Department of Public Health, Nova Southeastern University, 3200 South University Dr., Health Professions Division, Ft. Lauderdale, FL 33328, USA; 2 The ElderCare Companies, Inc, 2517 State Rt. 35, Bldg. J Ste. 203, Manasquan, NJ 08736, USA; 3 College of Pharmacy, Nova Southeastern University, 3200 South University Dr., Health Professions Division, Ft. Lauderdale, FL 33328, USA

## Abstract

**Background:**

Falls among the elderly are a major public health concern. Therefore, the possibility of a modeling technique which could better estimate fall probability is both timely and needed. Using biomedical, pharmacological and demographic variables as predictors, latent class analysis (LCA) is demonstrated as a tool for the prediction of falls among community dwelling elderly.

**Methods:**

Using a retrospective data-set a two-step LCA modeling approach was employed. First, we looked for the optimal number of latent classes for the seven medical indicators, along with the patients’ prescription medication and three covariates (age, gender, and number of medications). Second, the appropriate latent class structure, with the covariates, were modeled on the distal outcome (fall/no fall). The default estimator was maximum likelihood with robust standard errors. The Pearson chi-square, likelihood ratio chi-square, BIC, Lo-Mendell-Rubin Adjusted Likelihood Ratio test and the bootstrap likelihood ratio test were used for model comparisons.

**Results:**

A review of the model fit indices with covariates shows that a six-class solution was preferred. The predictive probability for latent classes ranged from 84% to 97%. Entropy, a measure of classification accuracy, was good at 90%. Specific prescription medications were found to strongly influence group membership.

**Conclusions:**

In conclusion the LCA method was effective at finding relevant subgroups within a heterogenous at-risk population for falling. This study demonstrated that LCA offers researchers a valuable tool to model medical data.

## Background

Latent Class Analysis (LCA) is a statistical method for finding subtypes of related cases (latent classes) from multivariate categorical data [1]. The most common use of LCA is to discover case subtypes (or confirm hypothesized subtypes) based on multivariate categorical data [1-4]. LCA is well suited to many health applications where one wishes to identify disease subtypes or diagnostic subcategories [1-4]. LCA models do not rely on traditional modeling assumptions (normal distribution, linear relationship, homogeneity) and are therefore, less subject to biases associated with data not conforming to model assumptions [1-4]. In this paper, we demonstrate the utility of LCA for the prediction of falls among community dwelling elderly.

Falls among the elderly are a major public health concern. Research on falls and fall-related behavior among the elderly has found that falls are the leading cause of injury deaths among individuals who are over 65 years of age [5-11]. Research has shown that sixty percent of fall-related deaths occur among individuals who are 75 years of age or older [5-11]. Demography research estimates that by 2030, the population of individuals who are 65 years of age or older will double and by 2050 the population of individuals who are 85 years of age or older will quadruple [5-11].

Predicting elderly falling can be complex and often involves heterogeneous markers. Therefore, the identification of more homogeneous subgroups of individuals and the refinement of the measurement criteria are typically inter-related research goals. Appropriate statistical applications, such as latent class analysis, have become available for researchers to model the complex heterogenous measurements.

Latent class models are used to cluster participants. This type of model is adequate if the sample consists of different subtypes and it is not known before-hand which participant belongs to which of the subtypes [2]. The latent categorical variable is used to model heterogeneity. In the classic form of the latent class model, observed variables within each latent class are assumed to be independent, and no structure for the covariances of observed variables is specified [2].

LCA is one of the most widely used latent structure models for categorical data [12]. LCA differs from more well-known methods such as K-means clustering which apply arbitrary distance metrics to group individuals based on their similarity [13-15]. LCA derives clusters based on conditional independence assumptions applied to multivariate categorical data distributed as binomial or multinomial variables [16,17]. Using statistical distributions rather than distance metrics to define clusters helps in evaluating whether a model with a particular number of clusters is able to fit the data, since tests can be performed to observed (ni) versus model expected values (mi), using exact methods as recommended [18,19]. This comparison gives rise to a *χ*^2^ test of global model fit, in which significant values indicate lack of fit [20]. Here lack of fit means deviation of (model) predicted (m) frequencies from observed frequencies (n) [16].

Latent class analysis assumes that each observation is a member of one and only one latent class (unobservable) and that the indicator (manifest) variables are mutually independent of each other [20]. The models are expressed in probabilities of belonging to each latent class. For example, seven manifest variables can be expressed as:

$$\pi_{\mathrm{ijklmnot}}{=\pi }_{t}^{X}\pi_{\mathrm{it}}^{A|X}\pi_{\mathrm{jt}}^{B|X}\pi_{\mathrm{kt}}^{C|X}\pi_{\mathrm{lt}}^{D|X}\pi_{\mathrm{mt}}^{E|X}\pi_{\mathrm{nt}}^{F|X}\pi_{\mathrm{ot}}^{G|X}$$

where $\pi_{t}^{X}$ denotes the probability of being in a latent class (t = 1,2,…,T) of latent variable X; $\pi_{\mathrm{it}}^{A|X}$ denotes the conditional probability of obtaining the *i*th response from item A, from members of class t, i = 1,2,…,I; and $\pi_{\mathrm{jt}}^{B|X}\pi_{\mathrm{kt}}^{C|X}\pi_{\mathrm{lt}}^{D|X}\pi_{\mathrm{mt}}^{E|X}\pi_{\mathrm{nt}}^{F|X}\pi_{\mathrm{ot}}^{G|X}$, j = 1,2,…,j k = 1,2,…,k l = 1,2,…,l m = 1,2,…,m n = 1,2,…,n O = 1,2,…,O are the corresponding conditional probabilities for items B,C,D,E,F, and G respectively.

**Figure 1:**
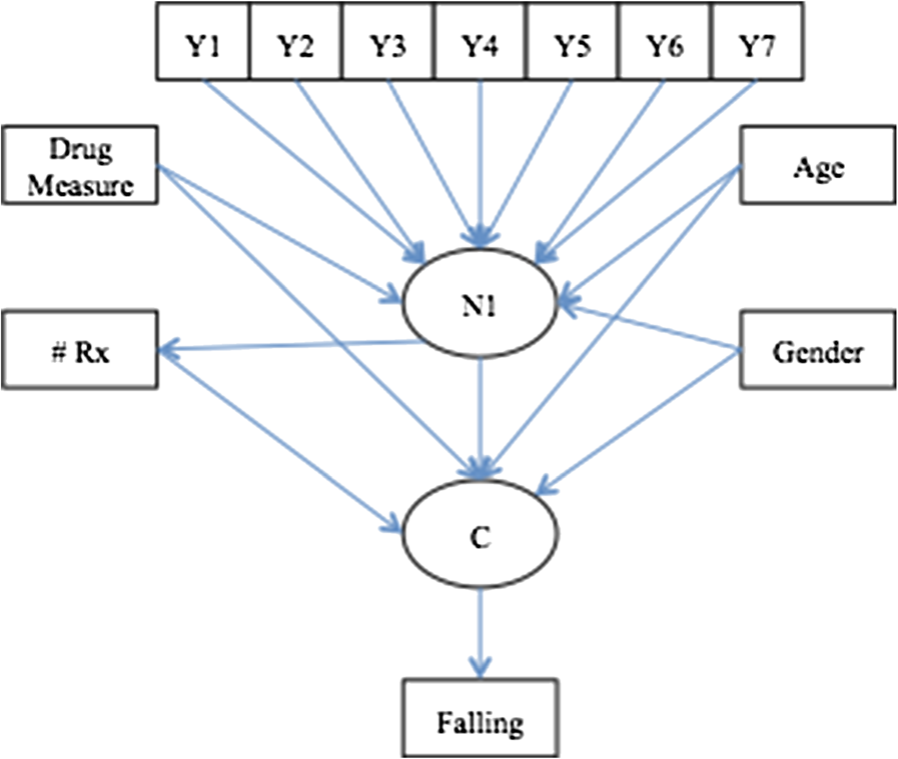
**Proposed fall model for the latent class analysis.** Y_i_ are the observed categorical medical indicators on the latent classes C. Drug Measure is the correspondence analysis derived drug score for each subject. Age is the age of patient. # Rx is the number of prescriptions taken by each subject. Gender is the subjects reported gender. Falling is the distal outcome.

We are testing the hypothesis that a two-class distal relationship (fall/no fall) can explain the relationship among the biomedical, pharmacological and demographic variables. Proper analysis of this data requires the understanding of two interdependent outcomes.^2^ First, the binary outcome is whether or not the event occurred (fall or no fall) and second what covariates increase or decrease the likelihood of this occurrence. The four specific aims of the study are to identify items that indicate classes, estimate class probabilities, relate the class probabilities to covariates, and predict a distal outcome (fall/no-fall) based on class membership. We model this process through the application of latent class analysis (Figure 1).

## Methods

A convenient retrospective database consisting of a random sample of 3,293 elderly patients was used to develop a model to predict the likelihood of falling among individuals aged 65 years or older. Due to the retrospective nature of this study, this study was granted an exemption in writing by the Nova Southeastern University’s IRB. This is a poof-of-concept analysis so it should be noted that this data set was not designed for an LCA, therefore, additional medical variables which may predict falling were not available. For this study an elderly person was defined as someone aged 65 years or older. Descriptive data were as follows (Table 1): The average age of patients was 77 years old; 32 percent of the subjects had fallen in the last 30 days; falling patients were taking an average of five prescription medications while non-fallers were consuming two; and 75 percent of the subjects were female. Research demonstrates that about 22% of community-dwelling elderly persons fall each year; 10% of these "fallers" have multiple episodes [21]. This research was approved by Nova Southeastern University's Institutional Review Board for human subjects research.

**Table 1:** Descriptive statistics

**Variable**	**Statistic**	**No-Fall**	**Fall**
		**N = 1906**	**N = 908**
Age	Mean ± SD	77.47 ± 6.91	77.98 ± 7.41
Number of Medications	Mean ± SD	2.30 ± 5.57	5.10 ± 10.10
Gender	Male	27%	22%
	Female	73%	78%

The data set was taken from the State of Florida’s Elder Affairs Office. All variables were physician diagnosed and recorded in an electronic dataset using appropriate ICD-9 codes. Variables included in the database were:

### Biomedical

•Arthritis—defined as a person diagnosed with osteoarthritis (OA) and/or rheumatoid arthritis (RA). Presence or absence of arthritis was based on responses to questions on the basis of ICD-9 714.0, 715.× -716.×, from both principal and secondary diagnosis fields within a patient record.

•High Blood Pressure (HBP)—defined as a person diagnosed with hypertension. HBP was identified on the basis of ICD-9 codes 401–405, from both principal and secondary diagnosis fields within a patient record.

•Diabetes—defined as a person diagnosed with diabetes mellitus. Diabetes was identified on the basis of ICD-9 codes of 250.0×–250.5× and 250.7×–250.9× from both principal and secondary diagnosis fields within a patient record.

•Heart Disease (HD)—defined as a person diagnosed with coronary artery disease. HD was identified on the basis of ICD-9 codes 414.0x, from both principal and secondary diagnosis fields within a patient record.

•Foot Disorders (FD)—defined as a person diagnosed with peripheral neuropathy, foot wounds, peripheral vascular disease, or Charcot arthropa. FD was identified on the basis of ICD-9 codes 356.9, 892.0-892.2, 443.9, and 713.5 from both principal and secondary diagnosis fields within a patient record.

•Parkinson’s Disease (PD)—defined as a person diagnosed with Parkinson’s Disease. PD was identified on the basis of ICD-9 code 332.0 from both principal and secondary diagnosis fields within a patient record.

•Stroke—defined as a person diagnosed with occlusion and stenosis of precerebral arteries including basilar artery, carotid artery, and vertebral artery, etc.; occlusion of cerebral arteries including cerebral thrombosis and Cerebral embolism; unspecified cerebral artery occlusion; and transient cerebral ischemia. Data from both principal and secondary diagnosis fields within a patient record.

### Pharmacological variables

•Type of prescription medication—type of prescription medication was taken from patient records.

•Number of prescription medications—was taken from patient records.

### Demographic variables

•Age—was taken from patient records.

•Gender—Self reported male or female taken from patients’ record.

### Outcome

•Falling—was defined as “an event which results in the person coming to rest inadvertently on the ground or other lower level, and other than as a consequence of sustaining a violent blow.” Falling was taken from both principal and secondary diagnosis fields within a patient record.

A two-step modeling approach was employed. First, it was necessary to reduce the number of different medications (N = 121). Initially, a licensed geriatric pharmacist (PharmD) reviewed the medication list for accuracy and to remove medications that have not been shown to impact the probability of falling. Using correspondence analysis (CA) the medications were converted to continuous scores. CA is an exploratory technique related to principal components analysis which finds a multidimensional representation of the association between the row and column categories of a multi-way contingency table [22]. This technique finds scores for the row and column categories on a small number of dimensions which account for the greatest proportion of the *chi*^*2*^ for association between the row and column categories, just as principal components account for maximum variance [22]. These scores were then used in the latent class analysis. Similar to other data reduction techniques, CA can be used to transform data [23].

Second, we looked for the optimal number of latent classes for the seven binary indicators: (1) arthritis, (2) high blood pressure, (3) diabetes, (4) heart disease, (5) foot disorders, (6) Parkinson’s disease, and (7) stroke; along with the patients’ medication “score” and three covariates (age, gender, and number of medications). The appropriate latent class structure, with the covariates, were modeled on the distal outcome (fall/no fall). The default estimator was maximum likelihood with robust standard errors. The Pearson chi-square, likelihood ratio chi-square, (BIC), Lo-Mendell-Rubin Adjusted Likelihood Ratio test and the bootstrap likelihood ratio test were used for model comparisons.

## Results

### Data reduction

Based on patient charts forty-one different medications were used in the latent class analysis (Table 2). To reduce this to a manageable number, correspondence analysis (CA) was employed and the CA values were saved for use in the latent class analysis. Due to missing data this reduced the number of subjects in the final model to 2,814. The higher the CA score the more likely the medication will induce a fall. CA scores are given by the following formula

$$D_{r}^{-0.5}\left(P-rc^{2}\right)D_{c}^{-0.5}$$

where:

•P is the matrix of counts divided by the total frequency

•r and c are row and column sums of P

•the Ds are diagonal matrices of the values of r and c

**Table 2:** List of medications and correspondence scores

**Level**	**Number**	**Score**
PHENOBARBITAL	8	−0.690
CLOMIPRAMINE	8	−0.690
METHADONE	7	−0.690
IMIPRAMINE	17	−0.423
MORPHINE	36	−0.423
PRIMIDONE	43	−0.405
HYDROCODONE	46	−0.262
DIAZEPAM	128	−0.234
CHLORDIAZEPOXIDE	45	−0.225
RBAMAZEPINE	37	−0.187
OXAZEPAM	23	−0.155
MIRTAZAPINE	86	−0.124
AMITRIPTYLINE	180	−0.095
ALPRAZOLAM	1297	−0.094
CLONAZEPAM	368	−0.077
BUSPIRONE	124	−0.072
OXYCODONE	206	−0.054
GABAPENTIN	210	−0.051
DIGOXIN	272	−0.038
MEPROBAMATE	44	−0.010
LORAZEPAM	572	0.009
DISOPYRAMIDE	9	0.023
NEFAZODONE	6	0.023
PHENYTOIN	35	0.023
TEMAZEPAM	1470	0.030
ESTAZOLAM	179	0.045
PAROXETINE	338	0.049
CHLORPROMAZINE	11	0.165
TRIAZOLAM	16	0.227
FLUOXETINE	310	0.241
BACLOFEN	25	0.379
DESIPRAMINE	6	0.379
HYDROMORPHONE	7	0.379
HALOPERIDOL	11	0.379
CITALOPRAM	60	0.414
TRAZODONE	234	0.455
BUPROPION	20	0.498
DOXEPIN	83	0.580
PERPHENAZINE	19	0.593
AMOXAPINE	7	1.449
THIORIDAZINE	8	1.449

**Figure 2:**
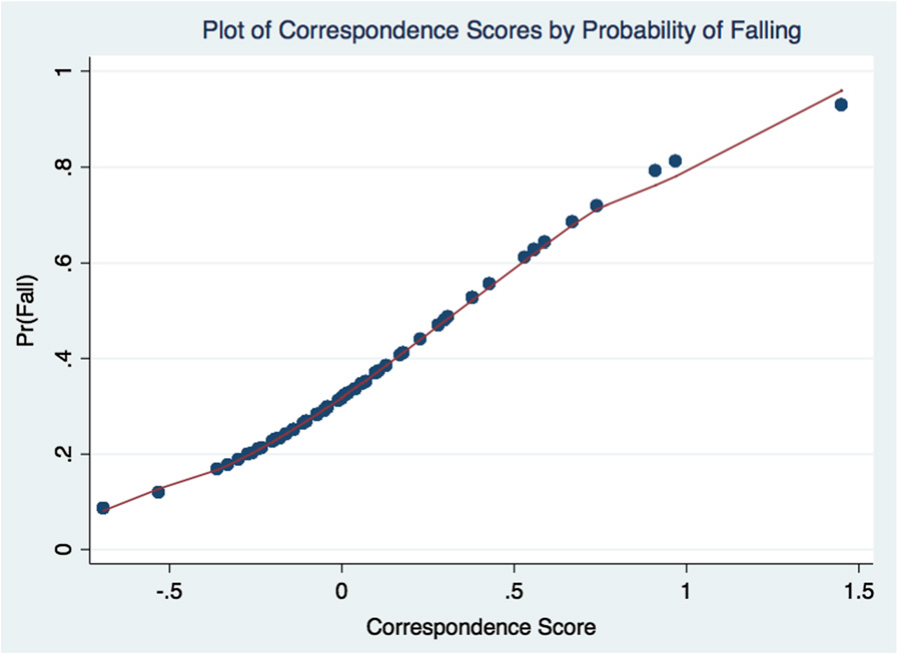
**Proposed fall model for the latent class analysis.** This is a plot of the probability of falling by correspondence analysis derived drug score.

A plot of the values indicates that an elderly person with a score of 0.40 has a 50% chance of falling (Figure 2). For this manuscript CA values are averaged for each person and referred to as the drug falling measure (singular value = 25%, inertia = 6% chi-square = 164.63). For example, a person may be using three different medications with CA values of -0.30, 0.20, and 1.20; so their drug falling measure is 0.37. A higher drug falling measure is associated with a higher probability of falling (*r* = .19, *p* < 0.00).

### Latent class analysis

**Figure 3:**
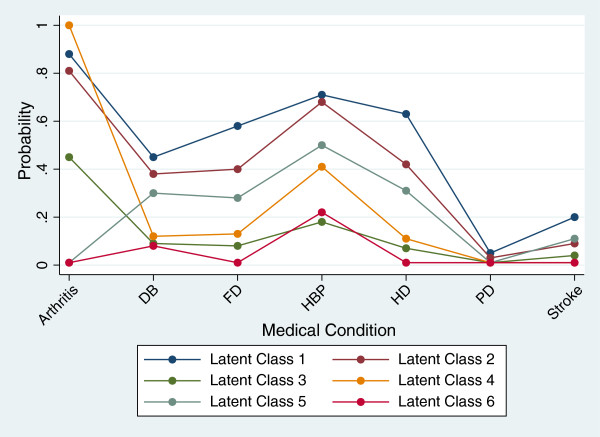
**Overlay plot of latent classes by medical condition.** Arthritis = Arthritis. HBP = High Blood Pressure. DB = Diabetes. HD = Heart Disease. FD = Foot Disorders. PD = Parkinson’s Disease. Stroke = Stroke.

For the latent class analysis, a review of the model fit indices shows that a six-class solution was preferred (Table 3). The six-class solution provided a lower Bayesian Information Criteria--BIC (lower is better), much smaller chi-square values, and as indicated by the procedures (Lo-Mendell-Rubin likelihood ratio test--LMR and bootstrap likelihood ratio test--BLRT), non-significant p-values. Age, number of medications, and gender were shown to have a significant impact on falling. Females, older patients, and the more prescription drugs an elderly person takes, the greater the probability that they will fall. Table 2 provides a comparison of fit indices for four-class, five-class, six-class and seven-class solutions. The six class structure, with covariates is interpreted as follows:

•Class one is most likely to be affected by all medical conditions (Figure 3). The average age of this class is 77.78 ± 7.01, the average number of medications is 4.7, and the average drug falling measure is 0.016. Latent class one is defined as the Poorest-Health Group I. Seventeen percent of the sample is classified into latent class one (Table 4). The classification accuracy is 95%; the misclassified elderly were all placed into class four (Table 4). Subjects in class one have a 47% chance of falling. The odds ratio indicate that a person in class one is 4.41 times more likely to fall than a person in class six: Healthy Group II (Tables 5 and 6).

•Class two is also affected by all measured medical conditions (Figure 3). The average age of this class is 76.89 ± 7.02, the average number of medications is 7.5, and the drug falling measure is 0.017. This is defined as the Poorest-Health Group II. Twenty-eight percent of the sample is placed into latent class two (Table 4). The classification accuracy is 89% (Table 4); misclassified elderly were placed into class three. Subjects in class two have a 46% chance of falling. The odds ratio indicate that a person in class two is about 4.67 times more likely to fall than a person in class six: Healthy Group II (Tables 5 and 6).

•Class three is generally unaffected by all medical markers (Figure 3). The average age of this class is 78.83 ± 6.63, the average number of medications is 7.8, and the drug falling measure is 0.006. We define this as the Healthy Group I. Seventeen percent of the sample is classified class three (Table 4). The classification accuracy for latent class three is 84% (Table 4). Misclassified elderly were placed into class two, indicating some overlap between the two latent classes. Subjects in class three have a 16% chance of falling. There is no significant difference in the likelihood of falling between class three and class six: Healthy Group II (Tables 5 and 6).

•Class four is primarily affected by arthritis; therefore, this is defined as the arthritis group (Figure 3). Twenty-percent of the sample fell into latent class four (Table 4). The average age of this class is 78.69 ± 7.32, the average number of medications is 2.6, and the drug falling measure is -0.003. The classification accuracy is 96% (Table 4). Misclassified elderly were placed into class one. Subjects in class three have a 26% chance of falling. The odds ratio indicate that a person in class four is approximately 2.07 times more likely to fall than a person in class six: Healthy Group II (Tables 5 and 6).

•Class five is primarily affected by high blood pressure,diabetes, heart disease and foot disorders (Figure 3). This group is defined as the diabetes-heart disease group. Eight percent of the sample fell into latent class five (Table 4). The average age of this class is 77.53 ± 7.04, the average number of medications is 3.1, and the drug falling measure is -0.009. The classification accuracy is 95% (Table 4). Misclassified elderly were placed into either class one (Unhealthy Group I) or six (Healthy Group I). Subjects in class five have a 29% chance of falling. The odds ratio indicates that a person in class five is 2.24 times more likely to fall than a person in class six: Healthy Group II (Tables 5 and 6).

•Class six is least affected by the medical conditions and is defined as healthy group II (Figure 3). Ten percent of the sample fell into latent class six (Table 4). The average age of this class is 78.87 ± 7.48, the average number of medications is 4.3, and the drug falling measure is -0.012. The classification accuracy is 97% (Table 4). Subjects in class three have a 15% chance of falling. Misclassified elderly were placed into class five: the diabetes-heart disease group (Tables 5 and 6).

**Table 3:** Basic latent class structure

	**Four class solution**	**Five class solution**	**Six class solution**	**Seven class solution**
Pearson *χ*^2^	2519	2196	2173	2172
LR *χ*^2^	1171	1156	1143	1107
*χ*^2^*df*	478	469	462	454
Loglikelihood	−12922	−12012	−11672	−11509
Number of parameters	48	61	74	87
BIC	24226	23893	23718	23710
LMR (*p* value)	.000	.029	.758	.626
BLRT (*p* value)	.000	.028	.758	.626
Entropy	.854	.883	.893	.839

**Table 4:** Most likely latent class membership

	**Count**	**Proportion**	**Class 1**	**Class 2**	**Class 3**	**Class 4**	**Class 5**	**Class 6**
Class 1	477	17%	0.95	0.00	0.00	0.04	0.00	0.00
Class 2	792	28%	0.00	0.89	0.11	0.00	0.00	0.00
Class 3	486	17%	0.00	0.16	0.84	0.00	0.00	0.00
Class 4	553	20%	0.04	0.00	0.00	0.96	0.00	0.00
Class 5	222	8%	0.01	0.00	0.00	0.00	0.95	0.04
Class 6	284	10%	0.00	0.00	0.00	0.00	0.03	0.97
Total	2,814	100%						

**Table 5:** Most likely latent class membership

	**Class 1**	**Class 2**	**Class 3**	**Class 4**	**Class 5**	**Class 6**
Class 1	.95	.00	.00	.04	.00	.00
Class 2	.00	.89	.11	.00	.00	.00
Class 3	.00	.16	.84	.00	.00	.00
Class 4	.04	.00	.00	.96	.00	.00
Class 5	.01	.00	.00	.00	.95	.04
Class 6	.00	.00	.00	.00	.03	.97

**Table 6:** Odds ratios

**Class**		**Class**	**Odds ratio**	**P-Value**	**Lower 95% CI**	**Upper 95% CI**
6	Vs	1	4.41	0.000	3.07	6.46
6	Vs	2	4.67	0.000	3.32	6.72
6	Vs	3	1.12	0.574	0.75	1.69
6	Vs	4	2.07	0.000	1.43	3.04
6	Vs	5	2.24	0.000	1.45	3.49

Note: Base or comparison group is class six or the “healthy group II” group.

## Discussion

This paper demonstrated the utility of LCA in the measurement of falling among community-dwelling elderly. The basic idea underlying LCA is that variables differ across previously unrecognized subgroups [24]. These subgroups form the categories of a categorical latent variable. Given the potential for confounding among the study variables, latent class analysis holds great promise.

The six-class solution was statistically sound and provided a relatively straightforward interpretable number of classes. The interpretation of a LCA relies on both the statistical indices and the practical interpretation of the classes. In our example, the statistical indices strongly point toward a six factor model. The classification accuracy for the model was very good. Furthermore, we were able to define each latent class, which provides researchers and practitioners practical implications of the analysis.

Medication usage helped differentiate the latent classes. Subjects in latent class one have higher probabilities of possessing the seven medical conditions than subjects in latent class two; yet, subjects in latent class two possess similar rates of falling. This may be explained by the number of medications that class two is taking (7.5 vs. 4.7). Similarly latent class three and six are both defined as the healthy groups. Differentiating the two groups is the number of medications taken by subjects in latent class three vs. latent class six (7.8 vs. 4.3).

It also true that the type of medications subjects are taking is impacting their probability of falling. This can be demonstrated for latent class one. Holding age and number of medications at their means, females with a drug falling measure of 1.50 [i.e., Thioridazine & Amoxapine] have a 80% greater chance of falling than the same subjects with a drug falling measure of -0.50 [i.e., Imipramine & Methadone] (*p* < 0.05). We stress that the latent classes are composite variables, so one should not look at medications in isolation. As one would expect, the two latent classes with the highest probability of falling also possess the highest drug falling measure and the worst medical conditions.

## Conclusion

As was demonstrated in past research correspondence analysis is a useful tool for researchers examining prescription medication data [25]. Combining LCA with CA provides researchers a powerful tool for data reduction analysis. We demonstrated that this approach was effective for finding relevant subgroups with a heterogenous at-risk population for falling. Nevertheless, the results may not be relevant to other countries, with different lifestyles and different socio-economic status.

LCA and CA possess limitations which make its application to this type of modeling dependent on replication studies. The specific limitations include (1) Classes not known prior to analysis, and (2) Class characteristics not know until after analysis. Both of these problems are related to LCA being an exploratory procedure for understanding data. Furthermore, the items were not designed for a LCA approach. A latent class study designed a-priori may offer better solutions. We would also suggest that additional items (medical) be used which have demonstrated to impact falling among elderly community dwellers--such as eye disease and pain.

## Competing interests

The authors declare that they have no competing interests.

## Authors’ contributions

PH, DS and WH participated in the design, coordination, project planning and data collection. PH performed the statistical analysis and drafted the manuscript. All authors read and approved the final manuscript.

## Pre-publication history

The pre-publication history for this paper can be accessed here:

http://www.biomedcentral.com/1472-6947/13/60/prepub
